# Doxorubicin-Loaded Polyelectrolyte Multilayer Capsules Modified with Antitumor DR5-Specific TRAIL Variant for Targeted Drug Delivery to Tumor Cells

**DOI:** 10.3390/nano13050902

**Published:** 2023-02-27

**Authors:** Anastasia Gileva, Daria Trushina, Anne Yagolovich, Marine Gasparian, Leyli Kurbanova, Ivan Smirnov, Sergey Burov, Elena Markvicheva

**Affiliations:** 1 Shemyakin-Ovchinnikov Institute of Bioorganic Chemistry RAS, 117997 Moscow, Russia; 2 Laboratory of Bioorganic Structures, Shubnikov Institute of Crystallography of Federal Scientific Research Centre “Crystallography and Photonics” of Russian Academy of Sciences, 119333 Moscow, Russia; 3 Faculty of Biology, Lomonosov Moscow State University, 119192 Moscow, Russia; 4 Cytomed JSC, Orlovo-Denisovsky pr. 14, 197375 St. Petersburg, Russia

**Keywords:** polyelectrolyte multilayer capsules, antitumor protein DR5-B, targeted drug delivery, codelivery system, tumor spheroids, HCT-116 cells

## Abstract

Recently, biodegradable polyelectrolyte multilayer capsules (PMC) have been proposed for anticancer drug delivery. In many cases, microencapsulation allows to concentrate the substance locally and prolong its flow to the cells. To reduce systemic toxicity when delivering highly toxic drugs, such as doxorubicin (DOX), the development of a combined delivery system is of paramount importance. Many efforts have been made to exploit the DR5-dependent apoptosis induction for cancer treatment. However, despite having a high antitumor efficacy of the targeted tumor-specific DR5-B ligand, a DR5-specific TRAIL variant, its fast elimination from a body limits its potential use in a clinic. A combination of an antitumor effect of the DR5-B protein with DOX loaded in the capsules could allow to design a novel targeted drug delivery system. The aim of the study was to fabricate PMC loaded with a subtoxic concentration of DOX and functionalized with the DR5-B ligand and to evaluate a combined antitumor effect of this targeted drug delivery system in vitro. In this study, the effects of PMC surface modification with the DR5-B ligand on cell uptake both in 2D (monolayer culture) and 3D (tumor spheroids) were studied by confocal microscopy, flow cytometry and fluorimetry. Cytotoxicity of the capsules was evaluated using an MTT test. The capsules loaded with DOX and modified with DR5-B demonstrated synergistically enhanced cytotoxicity in both in vitro models. Thus, the use of the DR5-B-modified capsules loaded with DOX at a subtoxic concentration could provide both targeted drug delivery and a synergistic antitumor effect.

## 1. Introduction

Biodegradable polyelectrolyte multilayer capsules are promising for the entrapment of a wide range of various biologically active molecules due to the versatility and simplicity of the fabrication technique [1,2]. This technique allows to fabricate capsules under mild conditions avoiding any organic solvents and using a rather wide selection of shell materials, including biocompatible and biodegradable polymers [3,4,5,6,7]. Moreover, all interactions, including electrostatics, hydrophobic, hydrogen binding, covalent binding, DNA hybridization, stereocomplexation, specific recognition, etc., can be used to provide driving forces for multilayer shell assembly [8]. Intracellular delivery of bioactive species has great potential for clinical applications [1,3,4]. Furthermore, an LbL approach can be proposed for the fabrication of high-performance nanosystems, in order to combine several drugs in PMC. Two up-to-date reviews comprehensively summarize current strategies that enable advanced functionalization of polyelectrolyte capsules, their potential biomedical applications, challenges in manufacturing as well as further safe translation into the clinical [2,5]. Despite PMC usage for targeted drug delivery, most of the researchers have been focused on magnetic drug targeting [6,7,9,10,11,12]. There are only a few studies, where magnetic antibody-modified capsules have been proposed for targeted drug delivery [13,14].

As well known, the surface design of the microcapsules with antibodies can provide highly specific antibody–antigen interactions with simultaneous suppression of any unspecific binding [15,16]. For example, antibody-functionalized capsules assembled from poly(allylamine hydrochloride) and poly(acrylic acid) on 6 µm CaCO_3_ templates targeted major histocompatibility complex class I receptors into living cells [17]. The authors revealed that protein A functionalized polyelectrolyte microcapsules enhanced targeting efficiency by 40−50% over capsules with randomly attached antibodies via direct covalent coupling to the surface. Cortez et al. reported that non-degradable PMC from poly(sodium 4-styrenesulfonate) and polyallylamine hydrochloride were functionalized with the humanized A33 (HuA33) antibody in order to provide their binding to appropriate receptors overexpressed in the colorectal cancer cell line [18,19].

Other alternatives for the functionalization of the outer layer and the subsequent improvement of their targeting rely on the use of polysaccharides, in particular hyaluronic acid. Thus, some studies have been focused on the fabrication of capsules with an outer layer of hyaluronic acid, which allowed to enhance both the targeting and the internalization of the capsules by tumor cells [14,20]. The verification of cancer cell targeting and bio-adhesion abilities of hyaluronic acid was performed using shells of a combination of alginate, chitosan and hyaluronic acid assembled on 2 µm monodisperse spherical melamine formaldehyde resin particles [14] and poly-L-arginine, siRNA and hyaluronic acid polyelectrolytes adsorbed on 100 nm poly(lactic-co-glycolic acid) nanoparticles [20].

Tumor necrosis factor-related apoptosis-inducing ligand (TRAIL) is an antitumor cytokine, selectively targeting transformed cells, but not normal cells, upon binding to the DR4 and DR5 death receptors. However, a soluble wild type TRAIL did not demonstrate a sufficient antitumor activity in previous clinical trials, and therefore a list of modifications, including TRAIL immobilization on various nanocarriers, has been reported [21].

Recently, we have generated the DR5 receptor-selective TRAIL variant DR5-B, which can bind only to the DR5 death receptor, and, as a result, it can overcome a receptor-dependent resistance of tumor cells to TRAIL [22]. DR5 is considered the most important receptor for TRAIL apoptotic signaling [23]. Therefore, DR5-B can serve as a more specific ligand than TRAIL for tumor targeting.

Doxorubicin is widely employed in anticancer therapy due to its rather broad spectrum of antitumor activities. Presently, DOX is considered as a gold standard to treat several types of sarcomas, carcinomas, lymphomas, etc. However, like other anthracyclines, DOX is known to demonstrate irreversible cardiotoxicity which is related to the total cumulative dose to which the patient has been exposed to [24]. Nevertheless, due to its universal anticancer activity, DOX is still widely used in clinics. Therefore, the development of DOX-loaded capsules is of particular interest.

The aim of the current study was to fabricate biodegradable polyelectrolyte multilayer capsules loaded with doxorubicin and functionalized with the tumor-targeting DR5-B ligand, and to evaluate a combined antitumor effect of this targeted drug delivery system in vitro.

Here, for the first time, an influence of size and surface modifications of the nanosized DOX-loaded capsules fabricated by the LbL technique on cell uptake as well as on in vitro cytotoxicity was studied. The combination of antitumor activity of the DR5-B ligand with DOX (at a subtoxic concentration) loaded into the capsules allowed us to provide both targeted drug delivery and a synergistic antitumor effect of the DR5-B ligand and doxorubicin.

## 2. Materials and Methods

### 2.1. Materials

All chemicals were of analytical grade and used as received without further purification. Calcium chloride dihydrate (CaCl_2_·2H_2_O), anhydrous sodium carbonate (Na_2_CO_3_), glycerol, ethylenediaminetetraacetic acid (EDTA), dextran sulfate sodium (DS) salt (MW 50 kDa), poly-L-arginine hydrochloride (Parg) with MW 15–70 kDa, sodium chloride (NaCl), phosphate buffered saline (PBS), isopropyl-β-D-1-thiogalactopyranoside, Rhodamine 6G (MW 479), Fluorescein isothiocyanate (FITC-) dextran (MW 40 kDa), doxorubicin hydrochloride (MW 580) and Hoechst 33258 were purchased from Sigma-Aldrich; DMSO (dimethyl sulfoxide, 99.5%), Trypan Blue solution, phosphate-buffered saline (PBS, pH 7.4), 0.25% (*v*/v) trypsin-EDTA solution, Versene solution, Dulbecco’s modified Eagle’s medium with Phenol Red (DMEM) and MTT (Thiazolyl Blue Tetrazolium Bromide, 98%) were purchased from PanEko (Moscow, Russia). Fetal bovine serum (FBS) was obtained from PAA Laboratories (Pasching, Austria). Fluorescent dyes sulfo-Cyanine3 maleimide and BDP FL maleimide were obtained from Lumiprobe (Moscow, Russia). Deionized water from a three-stage Milli-Q Plus purification system was used in the experiments.

### 2.2. Expression and Purification of the Recombinant DR5-B Protein

The recombinant DR5-B protein was expressed and purified as previously described [25]. Briefly, the *E. coli* strain SHuffle B T7 was transformed with the pET32a/dr5-b plasmid vector, inoculated into the Terrific Broth (TB) medium containing ampicillin (100 μg/mL) and grown at 37 °C, 250 rpm for 5 h, followed by dilution (1:100) in the TB medium with ampicillin (100 μg/mL). At a cell optical density of 0.6, protein expression was induced by adding 0.05 mM isopropyl-β-D-1-thiogalactopyranoside solution, and the cells were grown overnight (250 rpm, 28 °C). Then, the cells were precipitated (5000 × g) at Beckman Coulter (USA) and stored at −80 °C. For protein purification, the cells were disrupted by a French press (Spectronic Instruments Inc., Irvine, CA, USA) under a pressure of 2000 psi, and DR5-B was purified from a soluble cytoplasmic fraction by metal-affinity chromatography on Ni-NTA agarose (Qiagen, Germantown, MD, USA), followed by ion exchange chromatography on SP Sepharose (GE Healthcare, Danderyd, Sweden). The purified protein was dialyzed against 5 mM Na_2_HPO_4_ (pH 7.0) and 150 mM of NaCl solutions and sterilized by filtration (Millipore, 0.22 µm). The protein concentration was determined by Bradford assay (Bio-Rad, Hercules, CA, USA). The expression level of the protein was analyzed using sodium dodecyl sulfate polyacrylamide gel electrophoresis (12% SDS-PAGE).

To obtain the fluorescently labeled protein DR5-B, the cysteine-modified protein DR5-B was obtained as described earlier [26]. Further, the cysteine-modified DR5-B protein was labeled by maleimide chemistry coupling either with fluorescent sulfo-Cyanine 3 maleimide or BDP-maleimide dye (Lumiprobe, Moscow, Russia) according to the manufacturer’s protocol.

### 2.3. Preparation of Polyelectrolyte Multilayer Capsules Loaded with Doxorubicin and Functionalized with the DR5-B Protein

The polyelectrolyte multilayer capsules were prepared by LbL technique using colloidal CaCO_3_ particles as a template. The CaCO_3_ particles of a submicron size were synthesized as described earlier [27]. In brief, equal volumes of 0.1 M calcium chloride and sodium carbonate salts were mixed with glycerol in a ratio of 1:1:5 (*v*/*v*/*v*). After stirring (500 rpm, 60 min), a suspension was centrifuged (12,000× *g*, 5 min) and the CaCO_3_ particles were washed three times with deionized water. Dextran sulfate and poly-L-arginine were used to obtain (Parg/DS)_3_ capsules comprising 3 bilayers, which were assembled on the surface of CaCO_3_ particles. DS and Parg were alternatively adsorbed from aqueous polymer solutions (2 mg/mL, 0.15 M NaCl), starting from the Parg layer. To dissolve CaCO_3_ cores, the obtained particles were treated with an EDTA solution (0.5 M, 5 mL) for 15 min. The obtained hollow multilayer capsules (mean size of approx. 500 nm) were divided into two parts, and one part of them was subjected to size minimization via thermal treatment as described earlier [28]. For this purpose, the capsule suspension was incubated at 90 °C for 60 min.

To visualize the capsules by confocal laser scanning microscopy (CLSM), submicron capsules were post-loaded with Rhodamine 6G. For this purpose, the aliquoted PMC suspension (200 µL) samples were mixed with a dye solution (0.01 mg/mL, 200 µL) for 15 min. For doxorubicin loading, the pre-formed (Parg/DS)_3_ capsules (in an amount of 10^10^ pieces) were re-suspended in a doxorubicin solution (1 mL, 0.02 mg/mL) and shaken for 60 min. Then the capsule samples were centrifuged (12,000× *g*, 5 min) to collect DOX-loaded capsules and then washed three times with deionized water.

To optimize the modification of the (Parg/DS)_3_ capsules with the DR5-B protein, an aliquot of capsules was varied in a range of 10^8^–3 × 10^10^ capsules in 250 µL, while a DR5-B amount was fixed (500 µL, 0.8 g/L). The aliquots of the capsule suspension were incubated with the aliquots of the DR5-B solution at stirring (500 rpm for 60 min). Then the capsules were separated by centrifugation (9000× *g*, 5 min) and washed twice with Milli Q water.

### 2.4. Characterization of the Polyelectrolyte Capsules

The doxorubicin loading efficiency was determined using UV-VIS PerkinElmer Lamdba C650 spectrophotometer. The absorbances of supernatant samples were detected at 485 nm wavelength (an absorption peak of doxorubicin). To calculate doxorubicin content in supernatants, a calibration curve was obtained based on doxorubicin solutions with previously known concentrations.

The concentrations of the remaining (non-adsorbed on the capsules) DR5-B protein in supernatants were determined by Bradford assay (Bio-Rad, Hercules, CA, USA) according to the manufacturer’s protocol. A similar protocol was used to modify PMC samples with the fluorescently labeled DR5-B protein.

The amount of loaded doxorubicin and DR5-B was expressed in the encapsulation efficiency (EE%), which was calculated by the following equation:

$EE\%=\frac{M_{initial}-M_{sup}}{M_{initial}}*100\%$, where $M_{initial}$ is the mass of doxorubicin/DR5-B in the solution before adding the capsule suspension, $M_{sup}$—is the mass of doxorubicin/DR5-B, which remained in the supernatant after encapsulation.

The drug loading capacity was calculated as the ratio of the weight of loaded DOX to the weight of CaCO_3_ particles × 100%.

The shape and surface morphologies of capsule samples were analyzed using a Jeol 7401F field emission scanning electron microscope (SEM). A secondary electron image was taken with a 5 kV electron beam at a working distance of 8.0 mm. The microcapsule suspension was washed with water and loaded on an SEM specimen stub. After drying overnight, the sample was sputter-coated with gold prior to SEM observation.

A surface charge (ζ-potential) and a hydrodynamic capsule size were measured by a Zetasizer ZS (Malvern Instruments, UK). Each ζ-potential value was averaged from three subsequent measurements (each of 15 runs), while a mean size was calculated from five subsequent measurements (each of 20 runs).

Confocal laser scanning microscopy was used to visualize the Rhodamine 6G-modified PMC. Confocal microphotographs were taken on Leica TCS SPE (Leica microsystems, Wetzlar, Germany) equipped with a 100× oil immersion objective.

To quantify the DOX release from PMC, a suspension of DOX-loaded capsules (10 vol. %) was resuspended in PBS (2 mL, pH 7.2) and incubated for 24, 36, 48, 60 and 72 h, respectively. Then the samples were centrifuged (14000 g, 5 min) and the supernatant was replaced with fresh PBS (pH 7.2). The concentration of released DOX in filler fluids was calculated using the following formula:

$R=100\%\;\times \;\frac{{OD}_{DOX}{}^{R}}{{OD}_{DOX}},$ where R is the drug release and %; ${\mathrm{OD}}_{\mathrm{DOX}}{}^{R}$ is an optical density of the released DOX measured at certain time points; ${\mathrm{OD}}_{\mathrm{DOX}}$ is an optical density of total DOX loaded into the capsules.

### 2.5. Cell Culture and Multicellular Tumor Spheroid Formation

Human colorectal carcinoma HCT-116 cells were cultivated in DMEM culture medium supplemented with 10% FBS in a humidified atmosphere (5% CO_2_) at 37 °C. The cells were detached after treatment with a trypsin-EDTA solution (0.25% *v*/*vv*), and the culture medium was replaced every 3–4 days. Multicellular tumor spheroids from HCT-116 cells were generated using a simple RGD-induced cell self-assembly technique, which has been recently developed by the authors of [29]. Briefly, cells (50,000 cells per 1 mL medium) were seeded in a 96-well culture plate (100 μL/well) and incubated at 37 °C (5% CO_2_) for 2–3 h until the cells became attached to the plate bottom. Then the medium was replaced in each well with 100 μL of complete DMEM (10% FBS) containing cyclo-RGDfK(TPP) peptide (40 μM). Finally, the cells were transferred to a CO_2_-incubator, and RGD-induced spheroid formation was observed in 72 h.

### 2.6. Confocal Microscopy

The cellular uptake of PMC and capsule localization were observed using confocal laser microscope (Nikon TE-2000, Japan). The cells or the tumor spheroids (40,000 cells per glass) were seeded on coverslips in complete DMEM (10% FBS) and transferred to the CO_2_–incubator to stay overnight. To visualize nuclei both in 2D (monolayer cell culture) and 3D (the tumor spheroids) in vitro models, the medium was replaced with DMEM containing a 50 μM Hoechst 33258 solution, and the cells or the tumor spheroids were incubated for 15 and 30 min, respectively, followed by washing with PBS (pH 7.4). Then, in the case of the 2D in vitro model, the cells were incubated either with the free DR5-B protein or with the capsules modified with DR5-B (where DR5-B was labeled with red fluorescent dye sulfo-Cyanine3 maleimide) or with unmodified PMC loaded with R6G rhodamine. All capsule samples were previously re-suspended in serum free DMEM (0.5 μL of the capsule suspension per mL of DMEM) in a CO_2_-incubator for 15 min and 1 h, respectively. In the case of the 3D in vitro model, the spheroids were incubated with unmodified PMC loaded with R6G rhodamine or with the DR5-B-modified capsules (where DR5-B was labeled with green fluorescent dye BDP FL maleimide) for 15 min and 1 h. Finally, the cells or the spheroids were washed three times with PBS (pH 7.4) and carefully transferred to the coverslips, fixed with a CC/Mount fluorophore protector and observed by confocal microscopy. The excitation/emission (λ_ex/em_) wavelength values were 525/548 nm for R6G rhodamine, 555/569 for sulfo-Cyanine3, 503/509 for BDP FL and 352/454 nm for Hoechst 33258.

### 2.7. Flow Cytometry and Fluorimetry

#### 2.7.1. Flow Cytometry

For flow cytometry analysis, a BD FACSCalibur fluorescent-activated flow cytometer with BD CellQuest software was used. The cells were seeded in a 24-well plate (50,000 cells/well) followed by overnight incubation (37 °C, 5% CO_2_). Then the culture medium was removed, and PMC loaded with DOX (0.5 μL of the capsule aliquot per 1 mL of the medium) was added to each well. After treatment, the cells were washed with PBS (pH 7.4) to remove non-internalized PMC. Flow cytometry data were expressed as median fluorescent intensity divided by the background intensity of the control (non-treated cells) after 30 min, 1, 1.5, 3 and 7 h incubation.

#### 2.7.2. Fluorimetry

To quantify the accumulation of free DOX, free DR5-B as well as PMC within the tumor spheroids, the samples previously re-suspended in DMEM were added to the spheroids in a 24-well plate (50,000 cells/well), and the plates were transferred to the CO_2_-incubator (37 °C, 5% CO_2_) for 1, 3 and 7 h, respectively. The samples were identified as DOX (free DOX), PMC_300_ + DOX (the 300 nm capsules loaded with DOX), PMC_300_-DR5-B + DOX (the samples, loaded with DOX and modified with DR5-B) and DR5-B (free DR5-B protein). Then the spheroids were washed with PBS (pH 7.4) and treated with DMSO; fluorescence was measured using a Promega GloMax-Multi detection system (USA) at 317 nm for excitation and 375–410 nm for emission. Uptake data were expressed as a percentage of fluorescence associated with the cells versus fluorescence of the feed solution.

### 2.8. Cytotoxicity Study In Vitro (MTT-Test)

The cells or the spheroids were seeded in a 96-well plate (7500 cells/well) followed by overnight incubation in a CO_2_-incubator (37 °C, 5% CO_2_). The unmodified PMC, free DR5-B, free DOX as well as the capsules loaded with DOX and/or with the DR5-B protein at various dilutions (0.1, 1, 10, 100 and 1000 ng/mL) were added to each well. The cells or the spheroids were transferred to the CO_2_-incubator for 24, 48 and 72 h, respectively. After treatment, the cells or the spheroids were incubated with an MTT-solution (0.05% *w*/*v*) in serum-free DMEM for 4 h. Then the medium was replaced with DMSO (100 μL/well), and an absorbance (570 nm) was measured using Multiskan FC reader (Thermo Scientific, USA). The half maximal inhibitory concentration (IC50) values were determined as a drug concentration, which resulted in 50% inhibition of cell growth.

### 2.9. Statistical Analysis

All data were normally distributed and were expressed as mean or mean ± SD. Statistical analysis of all results was performed using Student’s *t*-test. All experiments were carried out with at least three repetitions. Collected data were processed using GraphPad Prism and were accepted as significantly different when *p* < 0.05.

## 3. Results and Discussion

### 3.1. Preparation and Characterization of Polyelectrolyte Multicellular Capsules Loaded with Doxorubicin and Modified with the DR5-B Protein

**Figure 1:**
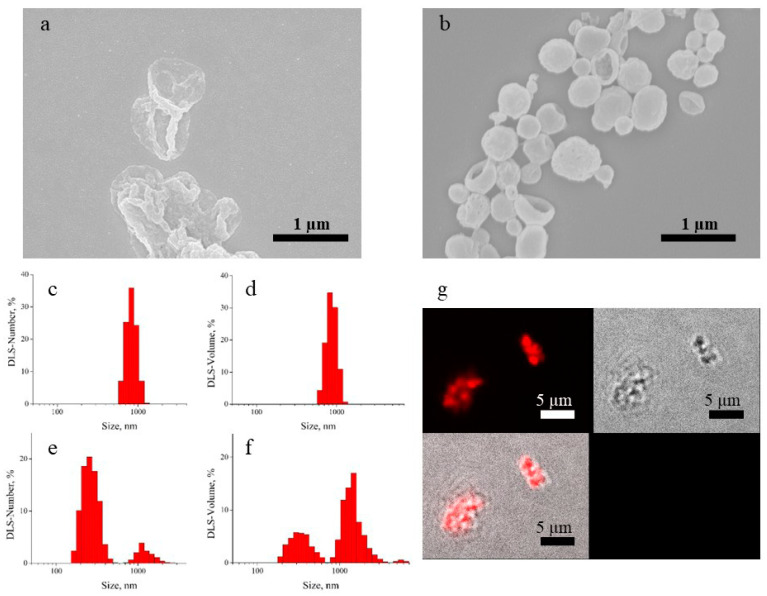
SEM images of the intact (**a**) and heat treated (**b**) (Parg/DS)_3_ capsules; DLS of the capsule samples: DLS-Number (**c**) and DLS-Volume (**d**) distributions for the intact capsules, DLS-Number (**e**) and DLS-Volume (**f**) distributions for heat treated capsules; CLSM images of the Rhodamine 6G-loaded intact (Parg/DS)_3_ capsules in fluorescent and transmission channels with their overlay (**g**).

In order to develop a targeted anticancer drug delivery system, a series of biodegradable polyelectrolyte multilayer capsules were obtained. An electrostatic interaction is a dominant driving force for oppositely charged DS and Parg to assemble a multilayer structure on the surface of vaterite submicron particles. In the current study, (Parg/DS)_3_ capsules comprising of three bilayers were obtained. Biodegradable PMCs were compacted according to the protocol of thermo-induced shrinking. After that, both intact and shrunken PMC were characterized in terms of their physico-chemical properties. Figure 1a,b reveals visible changes in the size and morphology of PMC after heat treatment, which follows from a rearrangement in the interpenetrating multilayers. The DLS-number distributions (Figure 1c,e) demonstrate an intense peak shift to the left as the result of heat treatment of the PMC suspension. As derived from the DLS-number data, the average hydrodynamic size of the heat-treated capsules was 320 ± 80 nm. The comparison of the DLS-volume data before and after heating (Figure 1d,f) shows that a significant amount of micron-sized objects remain in the sample after heating; aggregates probably remain as well. This can also be seen in the DLS-number distribution (Figure 1e) but in this case, the micron-sized peak is not very intense. It is worth mentioning that the number and volume distributions show a relative proportion of a number of differently sized objects as well as the volume occupied by them. As a result, a small amount of the aggregates or larger capsules can dominate in the DLS-volume distribution.

Successful Rhodamine 6G loading into the capsule cavity allowed us to visualize the intact capsules by confocal laser scanning microscopy (Figure 1g). Moreover, the capsules retain their aggregation stability when stored as an aqueous suspension at 4 °C for one month, which was confirmed by ζ-potential measurements. The ζ-potentials of the freshly prepared capsule samples and those ones after storage were –47 ± 6 and −42 ± 8 mV, respectively.

To provide DOX loading in a sub-toxic concentration, DOX encapsulation procedure was optimized. The encapsulation efficiency was found to be 85 ± 7%, which corresponded to approximately 0.002 pg of DOX per capsule.

**Figure 2:**
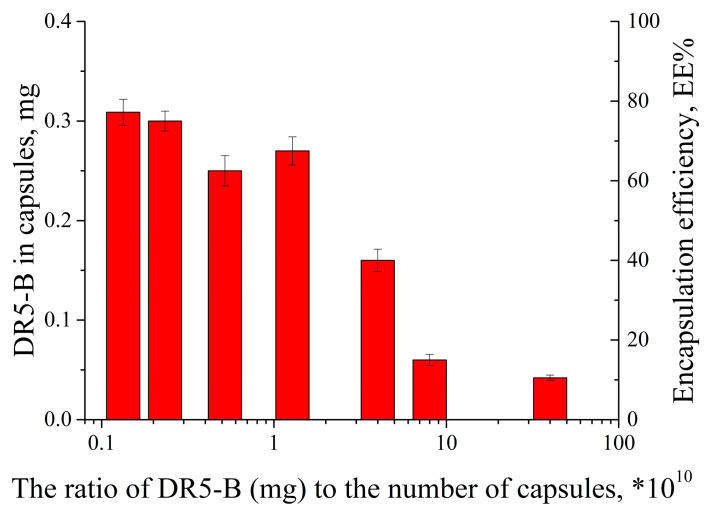
The DR5-B protein loading (mg) into the capsules and encapsulation efficiency (EE%) in the function of the DR5-B/a number of capsules ratio.

To optimize the surface modification of the capsules with the DR5-B protein, we carried out a series of experiments by loading a variable number of the capsules in a range of 1 × 10^8^–3 × 10^10^ with a fixed DR5-B amount. The amount of DR5-B loaded into the capsules was found to depend on the number of capsules incubated in the DR5-B solution (Figure 2).

An increase of the capsule number from 1 × 10^8^ to 2 × 10^10^ (in other words, decreasing the ratio of the DR5-B and the capsule number) resulted in an enhanced encapsulation efficiency from 12% to 81%. However, a further increase of the capsule number up to 3 × 10^10^ did not lead to a significant increase of the DR5-B protein loading. Therefore, we have optimized the conditions for efficient modification of the PMC surface with the DR5-B protein. Electrophoretic light scattering measurements showed that the modification of the PMC surface with DR5-B resulted in a change of the ζ-potential from a negative to a positive value (Supporting information, Figure S1a,b). Since the DR5-B molecules have regions that are charged differently (Supporting Information, Figure S1c), with an overall isoelectric point of about nine, the modification of the capsule surface by the protein is based on the electrostatic interaction of negatively charged sulfate groups and positively charged DR5-B units.

It has been shown that the amount of DR5-B protein released from the capsules at storage (4 °C, 4 weeks) was close to zero. Therefore, we could conclude that the DR5-B complex with the capsule shell was rather stable under these conditions.

**Figure 3:**
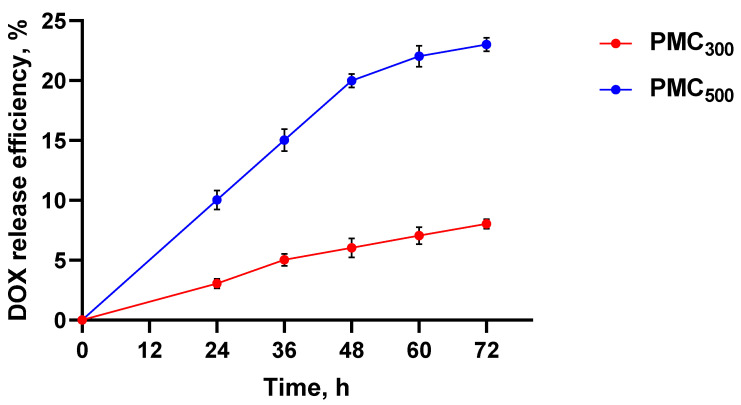
The DOX release efficiency from the PMC_500_ and PMC_300_. Dialysis in vitro.

Thus, it was of interest to compare two PMC types, namely untreated (here and further PMC_500_ according to the mean diameter) and heat-treated ones (here and further PMC_300_). The DOX release efficiency from PMC_500_ and PMC_300_ is shown in Figure 3. It is clearly seen that the DOX release from the untreated PMC_500_ was significantly higher than that of thermally compressed PMC_300_. Therefore, after 72 h, about 20% of the drug was released from the PMC_500_, while only 6% was released from the PMC_300_. For this reason, the PMC heat treatment could be used to reduce the drug release efficiency and, consequently, to ensure its sustained release.

### 3.2. Modification of PMC with Fluorescently Labeled DR5-B

To study an entrapment of the DR5-B protein, we used the modified DR5-B with the amino acid substitution of valine 114 to cysteine (DR5-B/V114C). It was previously designed for the covalent conjugation with polymer-based micellar nanocarriers, as the sulfhydryl group of cysteine residue allows it to conjugate with maleimide groups of various compounds by click chemistry [26]. In this study, DR5-B/V114C was conjugated with fluorophores, namely either sulfo-Cyanine3 maleimide (DR5-B/V114C-Cy3) or BDP FL maleimide (DR5-B/V114C-BDP), to compare fluorophore accumulation efficiency and localization both within the cells alone or those entrapped in PMC. The introduced V114C substitution, as well as the fluorescent label, did not affect the DR5-B ability to induce apoptosis (see Supplementary materials, Figure S2). As seen in Figure S2, for a monolayer culture of HCT-116 cells the cytotoxic activity of free DR5-B, DR5-B/V114C and DR5-B/V114C-Cy3 were identical.

### 3.3. Study of Capsule Accumulation and Localization in the Cells

The capsule accumulation in the cells was evaluated in two in vitro models, namely, using the monolayer cell culture (2D in vitro model) and the multicellular tumor spheroids (3D in vitro model). For this purpose, confocal microscopy, flow cytometry and fluorimetry were used.

#### 3.3.1. Study of Cellular Uptake of the Capsules by Confocal Microscopy

**Figure 4:**
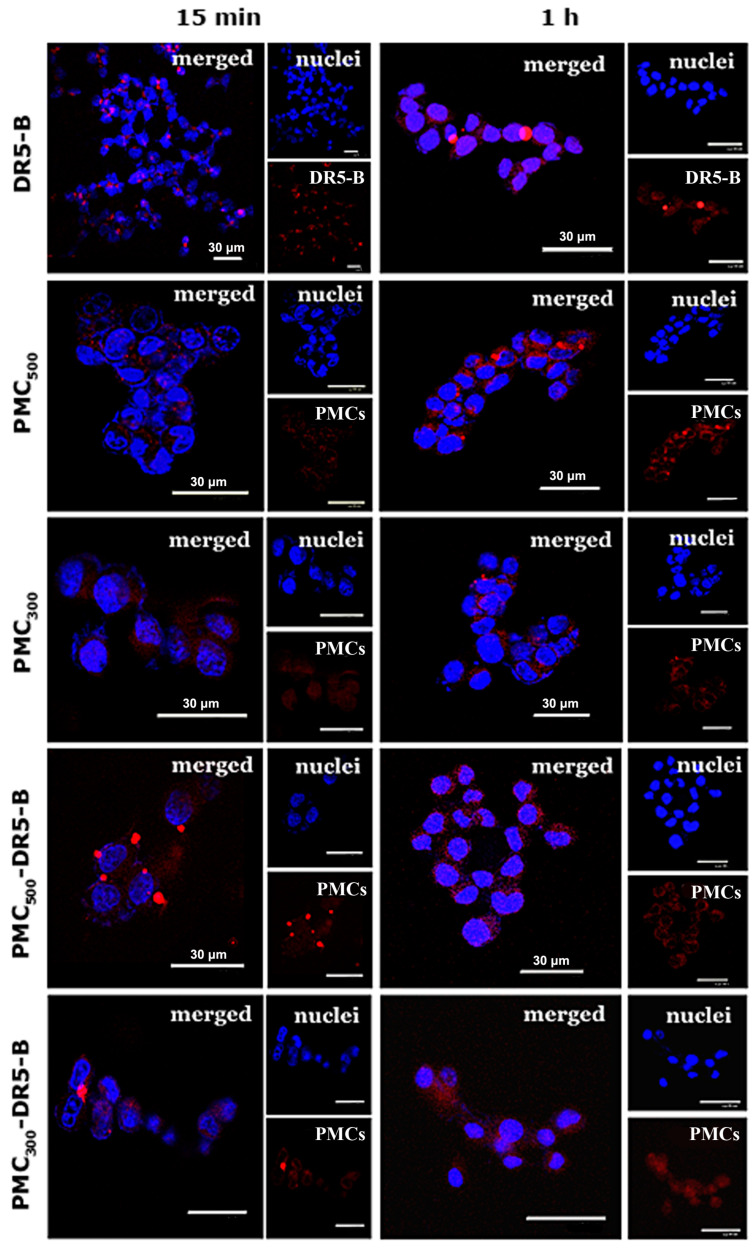
CLSM images of human colorectal carcinoma HCT-116 cells (monolayer cell culture) after 15 min and 1 h incubation with the free DR5-B protein, the unmodified capsule samples (PMC_500_ and PMC_300_ without DR5-B) as well as with the capsules modified with DR5-B. Free DR5-B (labeled with sulfo-Cyanine3 maleimide), the PMC samples modified with DR5-B (labeled with sulfo-Cyanine3 maleimide) and the unmodified PMC (loaded with R6G rhodamine) are in red. Cell nuclei stained with Hoechst 33258 are in blue. Confocal microscopy. The scale bar is 30 μm.

Qualitative analysis of the capsule accumulation and its intracellular localization was carried out by confocal laser microscopy (Figure 4). For this purpose, unmodified samples PMC_500_ and PMC_300_ loaded with R6G rhodamine dye, the PMC_500_ and PMC_300_ modified with the DR5-B protein and free DR5-B (as a control) were used. As seen from CLSM images, in the case of the monolayer cell culture (2D in vitro model) the free DR5-B protein was found to penetrate HCT-116 cells already after 15 min incubation, while in 1 h no significant fluorescence increase was observed.

The capsule samples PMC_500_ and PMC_300_, which were not modified with the DR5-B protein but contained the R6G Rhodamine dye, were also uptaken by the HCT-116 cells after 15 min incubation. However, their fluorescence intensity levels were only slightly higher than those of the free DR5-B protein. As for PMC_500_ and PMC_300_ modified with DR5-B, after 15 min incubation their accumulation levels within the cells were obviously higher than those of the unmodified capsules. In addition, after 1 h, the fluorescence levels of the PMC_500_ and PMC_300_ samples were markedly enhanced, which could be explained by the gradual accumulation of the capsules within the cells. It should be noted that the capsules were mainly localized around the nuclei and in the cell cytoplasm. On the other hand, the capsules modified with DR5-B were adsorbed on the cytoplasmic membrane after 15 min and were mainly localized in the cytoplasm, but not in the nuclei. After 1 h, the fluorescence intensity levels of the PMC_500_ and PMC_300_ modified with DR5-B were more or less similar.

The monolayer cell culture (2D in vitro model) is widely used to study cytotoxicity effects as well as to estimate capsule accumulation within the cells. However, the monolayer culture suffers from the lack of cell–cell interactions as well as the cell–matrix ones, and, therefore, it could not mimic in vivo conditions well. Nowadays there are several techniques that can be used to generate the multicellular spheroids that are a 3D in vitro model. They are considered to be a more adequate model for in vitro studies than monolayer cell culture. We have recently developed a simple universal approach based on RGD-induced cell self-assembly technique. This technique was recently used by us successfully to study cytotoxic effects of various nanocarriers loaded with anticancer drugs, for instance diethylaminoethyl dextran/xanthan gum nanocontainers loaded with thymoquinon [30], cerasomes loaded with DOX [31] and liposomes, for gene delivery [32].

**Figure 5:**
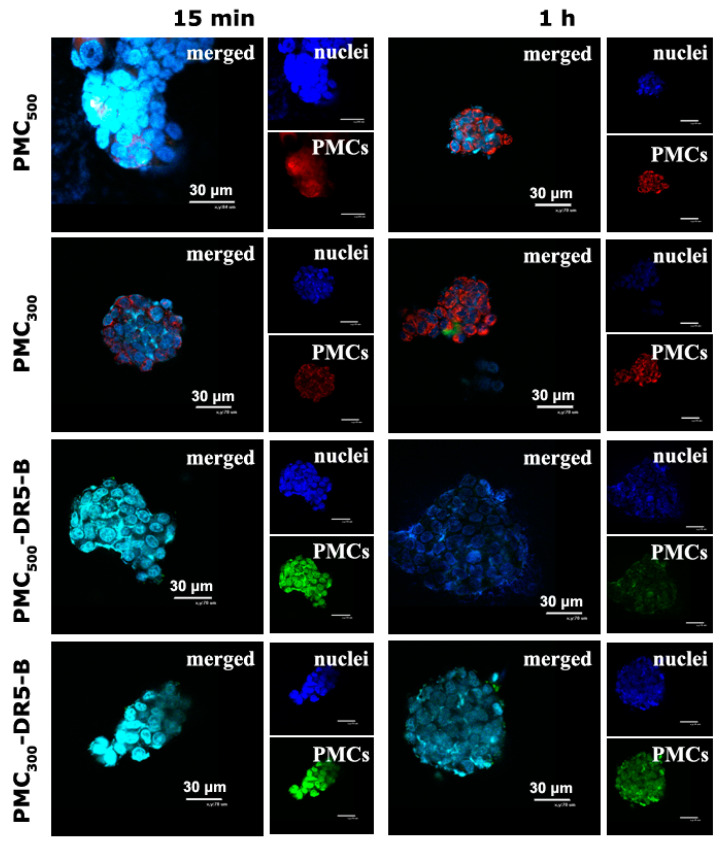
Spheroids from human colorectal carcinoma HCT116 cells (multicellular tumor spheroids) after 15 min and 1 h incubation with PMC_500_ and PMC_300_ samples. The unmodified PMC are stained with R6G rhodamine (in red) and the DR5-B is stained with a BDP FL maleimide dye (in green). Cell nuclei are stained with Hoechst 33258 (in blue). Confocal microscopy. The scale bar is 70 μm.

Thus, in the current study, a penetration and accumulation of PMC within the 3D multicellular tumor spheroids generated from HCT116 cells was also studied. As seen in Figure 5, after 15 min incubation unmodified PMC_500_ were observed within the spheroids, and in 1 h their fluorescence did not enhance. After 15 min incubation, the fluorescence intensity level of the unmodified PMC_300_ was a little bit lower than that of PMC_500_. However, after 1 h incubation the fluorescence values of both PMC_500_ and PMC_300_ were similar. As for DR5-B-modified PMC, there was no significant difference after 15 min and 1 h incubation.

#### 3.3.2. Study of Cellular Uptake of the Capsules by Flow Cytometry and Fluorimetry

Quantitative evaluation of accumulation efficiency was performed using flow cytometry for the 2D in vitro model and fluorimetry in the case of the 3D in vitro model. For this purpose, PMC_300_ and PMC_500_ loaded with doxorubicin (0.02 mg/mL, 85 ± 7% encapsulation efficiency) were used.

**Figure 6:**
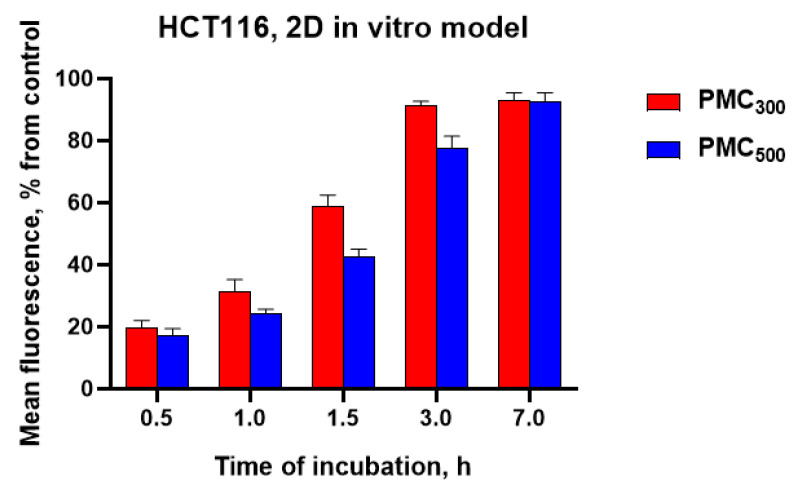
The accumulation levels of PMC_300_ and PMC_500_ loaded with DOX in HCT116 cells after 5, 30 and 60 min incubation. Monolayer cell culture. Flow cytometry.

First of all, it was of interest to evaluate a difference in accumulation efficacy levels of DOX-loaded PMC_300_ and PMC_500_ for monolayer cell culture (Figure 6). The obtained results revealed that PMC_300_ accumulation efficiency in HCT116 cells was higher than that for PMC_500_ at all time points (0.5, 1.0, 1.5, 3 and 7 h). For both capsule samples, the accumulation levels were growing with time. The PMC_300_ accumulation levels were 19, 31, 58, 89 and 90% after 0.5, 1, 1.5, 3 and 7 h incubation, respectively, while for PMC_500_ the appropriate values were 17, 22, 40, 76 and 89%. Thus, one could conclude that the smaller capsules PMC_300_ accumulated in the cells faster than the bigger ones (PMC_500_). Maximum accumulation of PMC_300_ was observed after 3 h incubation and it did not change in 7 h. As for PMC_500_, their accumulation value continued to gradually increase after 3 h and reached its maximum only after 7 h.

Tumor vessels are known to be a chaotic mixture of abnormal hierarchically disorganized vessels with many structural and functional differences from healthy tissues. In particular, such differences include the increased pore diameter of tumor blood vessels, characterized by increased permeability to nanoparticles, whose size does not exceed 400 kDa or 500 nm [33]. Thus, a number of studies have revealed that an optimal size of nanoparticles for antitumor drug delivery is within a range of 50–450 nm [34]. Moreover, an accumulation of the particles within the cells was found to be in function of the nanoparticle mean size: the smaller the particles were the higher the accumulation that was revealed [35,36,37].

**Figure 7:**
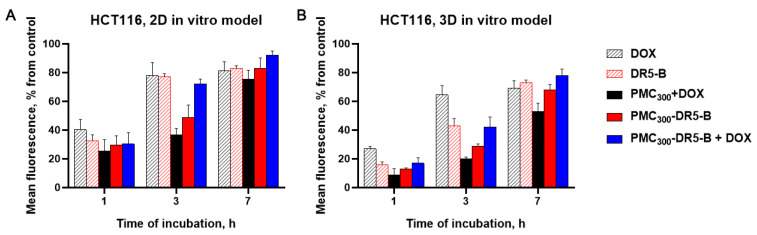
The accumulation efficiency levels of the capsules loaded with DOX (PMC_300_ + DOX), modified with DR5-B (PMC_300_-DR5-B), and the capsules loaded with DOX and modified with DR5-B (PMC_300_-DR5-B + DOX) in monolayer cell culture (**A**) and the tumor spheroids from HCT-116 cells (**B**) after 1, 3 and 7 h incubation. Free DOX and free DR5-B were used as controls. The results of flow cytometry (**A**) and fluorimetry (**B**).

In the current study, the DOX-loaded PMC_300_ were modified with DR5-B, in order to provide targeted DOX delivery to tumor cells together with achieving the synergistic antitumor effect. The PMC_300_ sample was chosen since it has shown the higher accumulation efficiency (see Figure 6). To study the cellular uptake of the capsules, flow cytometry (in case of 2D in vitro model) and fluorimetry (for 3D in vitro model) were used (Figure 7). Free DR5-B and PMC_300_-DR5-B samples were labeled with sulfo-Cyanine3 maleimide. The free DR5-B protein and free DOX were used as controls.

As seen in Figure 7, the maximum fluorescence levels in both 2D and 3D models were revealed for the capsules, which were loaded with DOX and modified with DR5-B. On the other hand, as was expected, the capsules accumulated in the spheroids more slowly than in the 2D in vitro model. This could be explained by the capsule penetration through several cell layers of the tumor spheroids. It should be noted that there was no significant difference in the accumulation of free DOX in 2D and 3D models, which could be related to rather low DOX molecular weight. A similar accumulation tendency for all samples was observed in both 2D and 3D in vitro models. Thus, in the case of the 2D model, for free DOX and free DR5-B samples the highest accumulation levels of 40 and 34%, respectively, were revealed after 1h. These values enhanced up to 78 and 77%, relatively, after 3 h. As for the spheroids, accumulations of both DOX and free DR5-B was slower in them than in the 2D model, namely 26 and 18% (in 1 h) and 63 and 43% (in 3h), respectively. The maximum accumulation levels of free DOX and the free DR5-B protein were revealed after 3 h incubation, in monolayer cell culture, while in tumor spheroids it took 7 h. This is in a good agreement with our previous results [30,31]. As for the capsule samples, the capsules loaded with DOX (PMC_300_+DOX) demonstrated the lowest accumulation efficiencies in all-time points both in 2D and 3D in vitro models. These values were 24, 38 and 77% (2D in vitro model) and 9, 19 and 53% (3D in vitro model) after 1, 3 and 7 h incubation, respectively. As for the capsule samples loaded with DOX and modified with the DR5-B protein (PMC_300_-DR5-B+DOX), their accumulation levels reached maximum values after 7 h in both 2D and 3D models. Therefore, as we expected, the modification of the DOX-loaded capsules with the DR5-B ligand provided their enhanced cellular uptake by HCT-116 cells.

### 3.4. Study of In Vitro Cytotoxicity Effects of the Capsules

**Figure 8:**
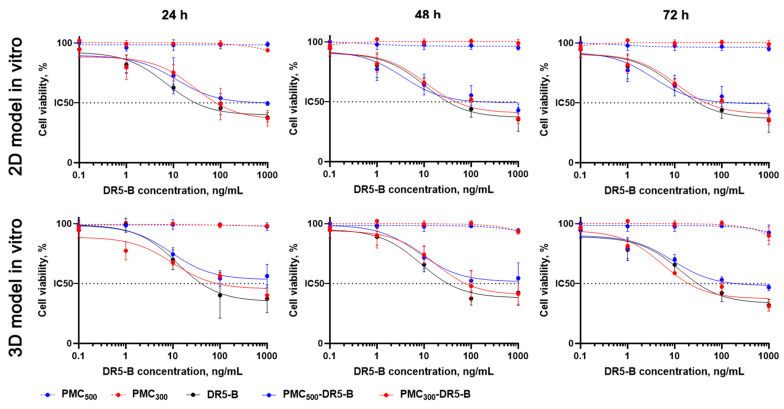
The cytotoxicity effects of the free capsules (PMC_300_ and PMC_500_) and the capsules modified with DR5-B (PMC_300_-DR5-B and PMC_500_-DR5-B) after incubation with human colorectal carcinoma HCT-116 cells after 24, 48 and 72 h incubation. Monolayer cell culture (a top panel) and the tumor spheroids (a lower panel). Free DR5-B protein was used as a control. MTT-test. *p* < 0.05 indicates significant difference according to Student’s *t*-test.

Cytotoxicity effects of the obtained DR5-B-modified PMC were evaluated by MTT-test (Figure 8, Table 1). In monolayer cell culture, the free DR5-B protein was the most cytotoxic in all time points, and IC50 values were 31 ± 2.6, 27 ± 3.2 and 10 ± 0.7 ng/mL after 24, 48 and 72 h, respectively. In contrast, PMC_300_ and PMC_500_ (without DR5-B) were completely non-toxic for the cells even after 72 h incubation with the highest sample concentration (IC50 > 1000 ng/mL). The DR5-B-modified capsules (PMC_300_-DR5-B) were more toxic than PMC_500_-DR5-B, which could be explained by their higher accumulation efficiency (see Figure 6). Thus, IC50 values were 72 ± 8.3 and 519 ± 38.5 ng/mL after 24 h, while after 72 h incubation appropriate values were 10 ± 3.4 and 24 ± 3.1 ng/mL for PMC_300_-DR5-B and PMC_500_-DR5-B, respectively.

As we expected, for all spheroids cytotoxicity values were lower than those for the monolayer cell culture. This could be explained by lower capsule penetration and accumulation within the cells in the 3D in vitro model due to a multilayer structure of the spheroids. These data are in good agreement with our previous results [29,32]. The free DR5-B protein was used as a control and demonstrated maximal cytotoxicity effects (see Table 1). The unmodified PMC_300_ and PMC_500_ were non-toxic (>1000 ng/mL). As seen in Table 1, the smaller capsules (PMC_300_-DR5-B) were more cytotoxic than the bigger ones (PMC_500_-DR5-B). For instance, after 72 h the IC50 values were 19 ± 1.6 and 109 ± 18.2 ng/mL for PMC_300_-DR5-B and PMC_500_-DR5-B samples, respectively. These results are in good agreement with our previously reported data obtained from the MCF-7 cells [38].

**Figure 9:**
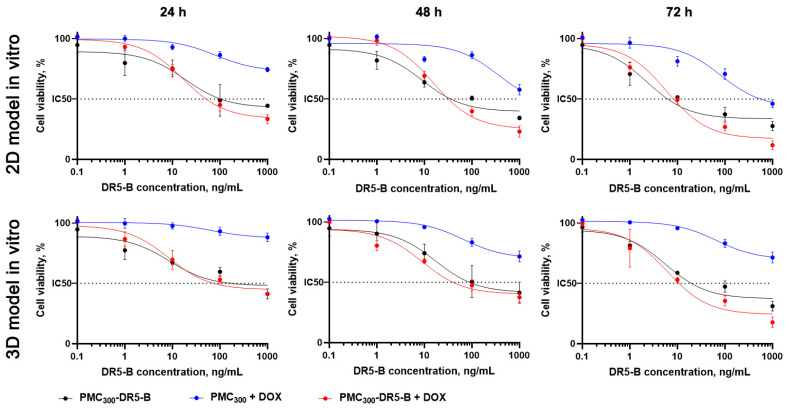
The cytotoxicity effects of the capsules modified with DR5-B (PMC_300_-DR5-B), the DOX-loaded capsules (PMC_300_ + DOX) and the DOX-loaded capsules modified with DR5-B (PMC_300_-DR5-B + DOX) on human colorectal carcinoma HCT-116 cells after 24, 48 and 72 h incubation. Monolayer cell culture (a top panel) and the tumor spheroids (a lower panel). MTT-test. *p* < 0.05 indicates significant difference according to Student’s *t*-test.

Thus, we have demonstrated that the DR5-B protein entrapped by PMC kept its cytotoxicity in tumor cells. Next, we aimed to investigate the cytotoxic effects of the capsules loaded with DOX and modified with DR5-B. Since the PMC_300_-DR5-B sample exhibited a higher antitumor effect compared to PMC_500_-DR5-B, it was selected for further experiments. The toxicity of the PMC_300_-DR5-B + DOX capsules was studied in both 2D and 3D in vitro models by MTT-test. The results are shown in Figure 9 and Table 2. Free DOX, free DR5-B, a combination of free DR5-B with free DOX (DR5-B+DOX) as well as hollow PMC_300_ capsules were used as controls.

As seen in Table 2, the IC50 values of PMC_300_-DR5-B+DOX were markedly lower than those of the PMC_300_-DR5-B sample. A similar effect was observed for both controls, namely free DR5-B and the combination of free DR5-B with free DOX. Thus, the capsules, which were loaded with DOX and modified with DR5-B (PMC_300_-DR5-B+DOX), demonstrated synergistically enhanced cytotoxicity in both 2D and 3D in vitro models. 

Previously, attempts have been made to modify a variety of nanosystems fabricated by various techniques with TRAIL for tumor targeting [21]. For instance, biodegradable nanoparticles based on poly(epsilon-caprolactone)-poly(ethylene glycol)-poly(epsilon-caprolactone) (PCL-PEG-PCL) loaded with curcumin were prepared by solvent emulsion evaporation technique and then coated with TRAIL protein via electrostatic interactions [39]. Another example is the gel-like PEGylated coacervate microdroplets (mPEG-Coa) loaded with TRAIL for the treatment of colon cancer. These microdroplets were fabricated from poly(ethylene arginyl-aspartate diglyceride) (PEAD) (i.e., polycation), which interacted with heparin (i.e., polyanionic counterpart) by electrostatic interaction in aqueous conditions, forming a gel-like Coa structure. TRAIL was simultaneously entrapped into the gel-like mPEG-Coa droplets [40]. In our study, we have used a simple and universal LbL technique for the fabrication of biodegradable multilayer capsules loaded with DOX and modified with the DR5-B, which is a DR5-specific TRAIL variant. The LbL technique has advantages over both the solvent emulsion evaporation method and the coacervation approach mentioned above. It allowed us to fabricate the hollow capsules (with mean sizes of 300 and 500 nm) with higher loading capacity compared to that of any polymer particles. Moreover, these capsules have potentially reduced immune response due to a variety of polymers for capsule preparation. Finally, the capsules modified with the DR5-selective TRAIL variant could be more advantageous over nanosystems containing a wild-type TRAIL due to DR5-B specific tumor targeting and the enhanced antitumor effect.

## 4. Conclusions

In this study, the biodegradable (Parg/DS)_3_ capsules modified with the DR5-B protein and loaded with DOX at subtoxic concentrations were prepared by the LbL technique. The capsules were characterized in terms of their mean size (500 and 300 nm after temperature treatment), ζ-potentials, DR5-B protein loading ability and DOX encapsulation efficacy. Cellular uptake of the capsules as a function of their size and surface modification with the DR5-B protein was evaluated by confocal microscopy, flow cytometry and fluorimetry using human colorectal carcinoma HCT-116 cells in both 2D and 3D in vitro models. The smaller capsules (PMC_300_-DR5-B) were found to be more cytotoxic than the bigger ones (PMC_500_-DR5-B) which was confirmed by MTT-test. As we expected, IC50 values in case of the spheroids (3D in vitro model) were higher than those for the monolayer cell culture (2D model). This could be explained by lower capsule penetration and accumulation within the cells due to a spheroid multilayer structure. The smaller capsules, which were loaded with DOX and modified with DR5-B (PMC_300_-DR5-B + DOX), demonstrated a 2–3-fold cytotoxicity enhancement compared to the capsules without DOX (PMC_300_-DR5-B) in both 2D and 3D in vitro models.

Thus, the developed DOX-loaded capsules modified with the DR5-B protein could be proposed as a novel targeted codelivery system, which was found to show an enhanced antitumor activity due to a rather high tumor targeting and synergistic effect of DR5-B and DOX. The inhibitory effect provided by the capsules containing subtoxic concentrations of DOX holds promise for reducing systemic toxicity while maintaining the therapeutic potential. This approach could be of great interest for the development of various nanodrugs for targeted anticancer therapy.

## List of Acronyms

DOXdoxorubicinDR4death receptor 4DR5death receptor 5DSdextran sulfate sodium salt (MW 50 kDa)FBSfetal bovine serumHCT-116human colorectal carcinoma cell lineLbLlayer-by-layerMTTthiazolyl blue tetrazolium bromide, 98%Pargpoly-L-arginine hydrochloride (MW 15–70 kDa)PMCpolyelectrolyte multilayer capsulesTB mediumTerrific Broth mediumTRAILtumor necrosis factor-related apoptosis inducing ligand

## Figures and Tables

**Table 1:** The IC50 values of the capsules after incubation with human colorectal carcinoma HCT-116 cells. Monolayer cell culture (2D in vitro model) and tumor spheroids (3D in vitro model). Free DR5-B protein was taken as a control. MTT-test.

Samples	IC50 * Values, ng/mL					
	2D In Vitro Model			3D In Vitro Model		
	24 h	48 h	72 h	24 h	48 h	72 h
**PMC_300_**	>1000					
**PMC_500_**						
**DR5-B**	31 ± 2.6	27 ± 3.2	10 ± 0.7	39 ± 4.0	33 ± 2.4	28 ± 5.1
**PMC_300_-DR5-B**	72 ± 8.3	42 ± 3.8	10 ± 3.4	96 ± 7.7	77 ± 5.2	19 ± 1.6
**PMC_500_-DR5-B**	519 ± 38.5	111 ± 10.9	24 ± 3.1	>1000		109 ± 18.2

* IC50 is half maximal inhibitory concentration.

**Table 2:** The IC50 values of the capsules on human colorectal carcinoma HCT-116 cells. Monolayer cell culture (2D in vitro model) and tumor spheroids (3D in vitro model). Free DR5-B protein, free DOX and the combination of free DR5-B with free DOX were taken as controls. MTT-test.

Samples	IC50 * Values, ng/mL					
	2D In Vitro Model			3D In Vitro Model		
	24 h	48 h	72 h	24 h	48 h	72 h
**PMC_300_**	>1000					
**DR5-B**	40 ± 5.8	31 ± 4.2	12 ± 2.2	56 ± 8.0	40 ± 8.8	31 ± 5.1
**DOX + DR5-B**	19 ± 2.4	7 ± 3.0	3 ± 0.9	53 ± 7.6	31 ± 4.3	10 ± 2.0
**DOX**	>1000	809 ± 31.3	665 ± 27.3	>1000		847 ± 65.3
**PMC_300_ + DOX**	>1000		593 ± 43.8	>1000		
**PMC_300_-DR5-B**	101 ± 9.6	38 ± 6.2	6 ± 1.2	107 ± 25.5	84 ± 10.4	19 ± 6.0
**PMC_300_-DR5-B+DOX**	52 ± 3.9	30 ± 3.7	7 ± 0.8	78 ± 8.1	47 ± 4.5	11 ± 3.5

* IC50 is half maximal inhibitory concentration.

## Data Availability

Not applicable.

## Supplementary Materials

The following supporting information can be downloaded at: https://www.mdpi.com/article/10.3390/nano13050902/s1, Figure S1: ζ-potential distributions for intact (Parg/DS)_3_ capsules (a), (Parg/DS)_3_ capsules after modification with targeted protein DR5-B (b), and for DR5-B protein molecules (c); Figure S2: Cytotoxicity of free DR5-B, DR5-B/V114C and DR5-B/V114C-Cy3 for colorectal carcinoma HCT-116 monolayer cell culture. MTT-test.
