# Supplementary material for: Doxorubicin-Loaded Polyelectrolyte Multilayer Capsules Modified with Antitumor DR5-Specific TRAIL Variant for Targeted Drug Delivery to Tumor Cells
Source: Nanomaterials (Basel). 2023 Feb 27;13(5):902. doi: 10.3390/nano13050902 (PMC10005140; doi:10.3390/nano13050902)
Supplement: Supplementary file 1 [file nanomaterials-13-00902-s001.zip › nanomaterials-2200794-supplementary.pdf]

nanomaterials

Supplementary Materials

Doxorubicin-Loaded Polyelectrolyte Multilayer Capsules Modified with Antitumor DR5-Specific TRAIL Variant for Targeted Drug Delivery to Tumor Cells

Anastasia Gileva [1,*,†], Daria Trushina [2,†], Anne Yagolovich [1,3,†], Marine Gasparian [1], Leyli Kurbanova [1], Ivan Smirnov [1], Sergey Burov [4] and Elena Markvicheva [1]

[1] Shemyakin-Ovchinnikov Institute of Bioorganic Chemistry RAS, 117997 Moscow, Russia
[2] Laboratory of Bioorganic Structures, Shubnikov Institute of Crystallography of Federal Scientific Research Centre “Crystallography and Photonics” of Russian Academy of Sciences, Moscow 119333, Russia
[3] Faculty of Biology, Lomonosov Moscow State University, 119192 Moscow, Russia
[4] Cytomed JSC, Orlovo-Denisovsky pr. 14, St. 197375 Petersburg, Russia,
* Correspondence: sumina.anastasia@mail.ru; Tel.: +79164517818
† These authors contributed equally to this work

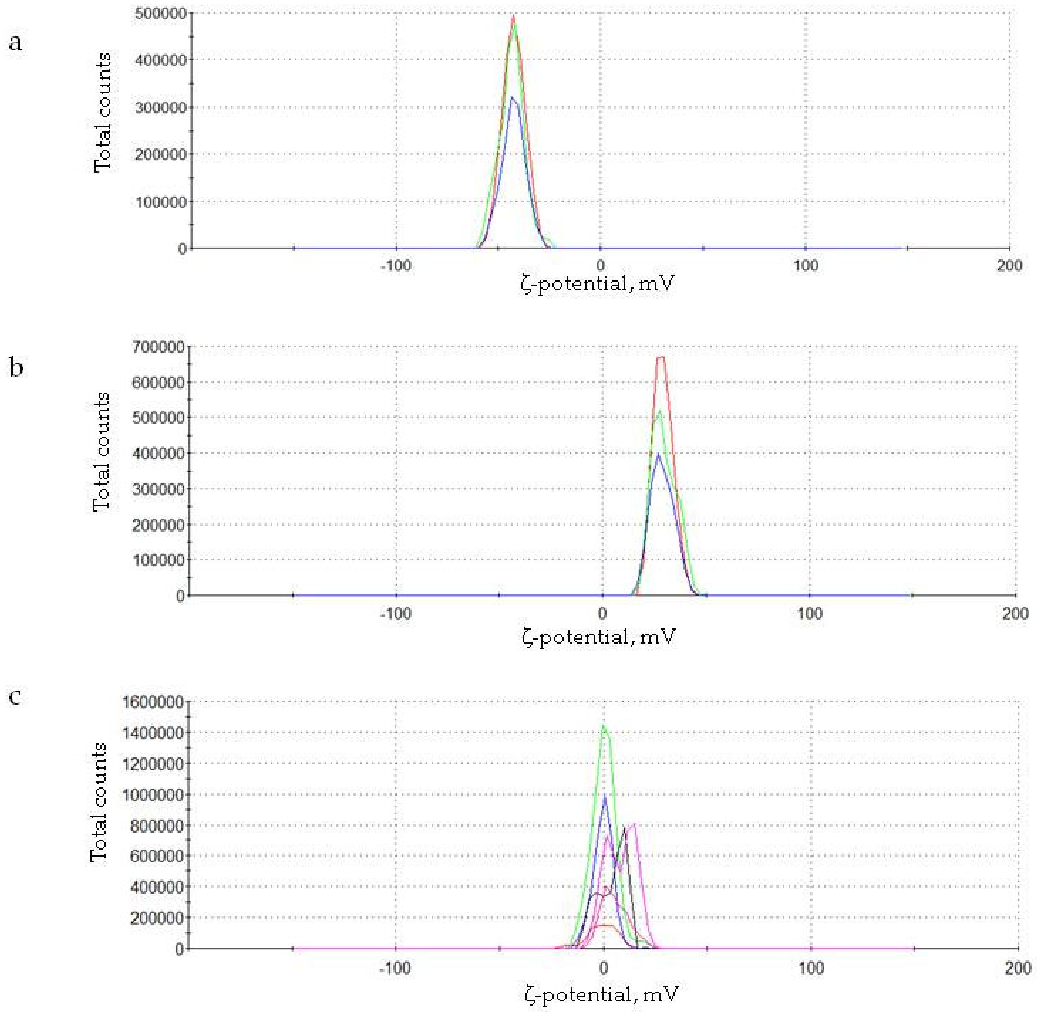

Figure S1. ζ-potential distributions for intact (Parg/DS)$_3$ capsules (a), (Parg/DS)$_3$ capsules after modification with targeted protein DR5-B (b), and for DR5-B protein molecules (c).

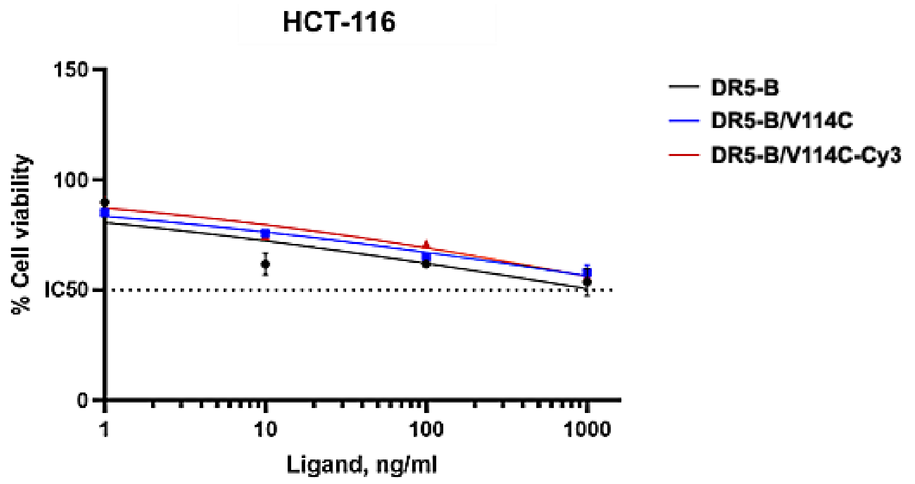

Figure S2. Cytotoxicity of free DR5-B, DR5-B/V114C and DR5-B/V114C-Cy3 for colorectal carcinoma HCT-116 monolayer cell culture. MTT-test.
